# Development and testing of a new application for measuring motion at the cervical spine

**DOI:** 10.1186/s12880-022-00923-1

**Published:** 2022-11-08

**Authors:** Koji Fujita, Kana Matsuo, Takafumi Koyama, Kurando Utagawa, Shingo Morishita, Yuta Sugiura

**Affiliations:** 1 Department of Functional Joint Anatomy, Graduate School of Medical and Dental Sciences, Tokyo Medical and Dental University, 1-5-45 Yushima, Bunkyo-Ku, Tokyo, 113-8519 Japan; 2 School of Science for Open and Environmental Systems, Graduate School of Science and Technology, Keio University, 2-15-45 Mita, Minato-Ku, Tokyo, 108-8345 Japan; 3 Department of Orthopaedic and Spinal Surgery, Graduate School of Medical and Dental Sciences, Tokyo Medical and Dental University, 1-5-45 Yushima, Bunkyo-Ku, Tokyo, 113-8519 Japan

**Keywords:** Cervical myelopathy, Image analysis, Motion measurement

## Abstract

**Background:**

Cervical myelopathy is a progressive disease, and early detection and treatment contribute to prognosis. Evaluation of cervical intervertebral instability by simple X-ray is used in clinical setting and the information about instability is important to understand the cause of myelopathy, but evaluation of the intervertebral instability by X-ray is complicated. To reduce the burden of clinicians, a system that automatically measures the range of motion was developed by comparing the flexed and extended positions in the lateral view of a simple X-ray of the cervical spine. The accuracy of the system was verified by comparison with spine surgeons and residents to determine whether the system could withstand actual use.

**Methods:**

An algorithm was created to recognize the four corners of the vertebral bodies in a lateral cervical spine X-ray image, and a system was constructed to automatically measure the range of motion between each vertebra by comparing X-ray images of the cervical spine in extension and flexion. Two experienced spine surgeons and two residents performed the study on the remaining 23 cases. Cervical spine range of motion was measured manually on X-ray images and compared with automatic measurement by this system.

**Results:**

Of a total of 322 cervical vertebrae in 46 images, 313 (97%) were successfully estimated by our learning model. The mean intersection over union value for all the 46-test data was 0.85. The results of measuring the CRoM angle with the proposed cervical spine motion angle measurement system showed that the mean error from the true value was 3.5° and the standard deviation was 2.8°. The average standard deviations for each measurement by specialist and residents are 2.9° and 3.2°.

**Conclusions:**

A system for measuring cervical spine range of motion on X-ray images was constructed and showed accuracy comparable to that of spine surgeons. This system will be effective in reducing the burden on and saving time of orthopedic surgeons by avoiding manually measuring X-ray images.

*Trial registration* Retrospectively registered with opt-out agreement.

**Supplementary Information:**

The online version contains supplementary material available at 10.1186/s12880-022-00923-1.

## Background

Cervical myelopathy is a neurological disease caused by age-related degeneration of the cervical spine and ossification of the posterior longitudinal ligament [1, 2]. When the disease progresses, numbness in the limbs, dyskinesia, gait disturbance, and vesicorectal disturbance appear [3, 4]; however, because the progression is relatively slow, patients are often unaware of their symptoms and are referred to a spine specialist only after the disease has become severe [5, 6]. The treatment of severe cervical myelopathy not only requires surgery but also has a worse prognosis than if therapeutic intervention is performed at an early stage [7]. Early diagnosis and treatment contribute to the prognosis.

**Fig. 1:**
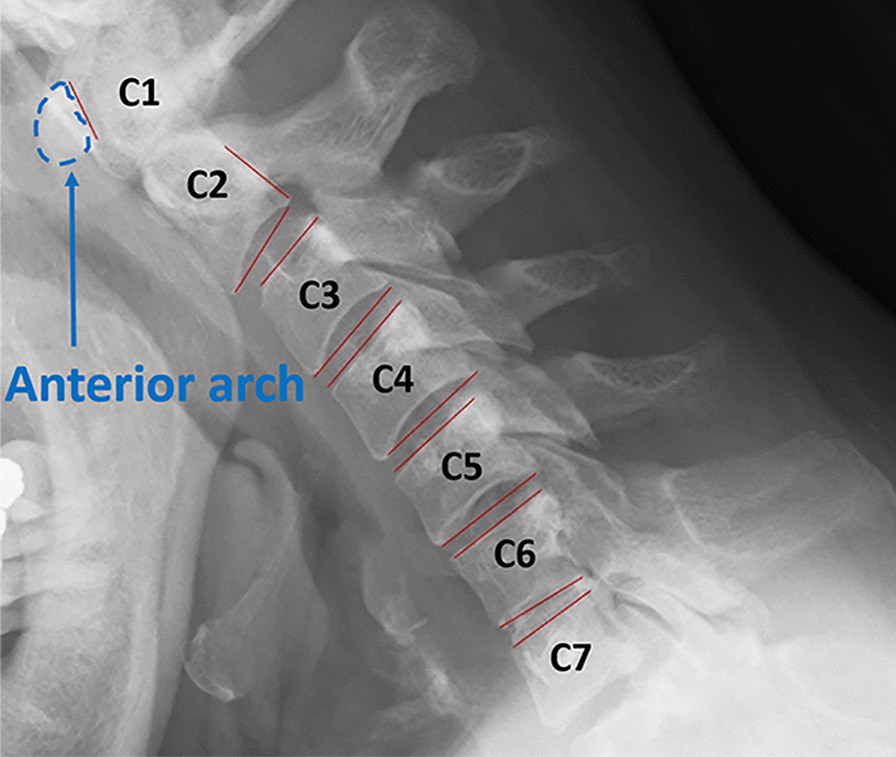
Example of annotation for measurement and cervical vertebrae call

The diagnosis of cervical myelopathy is based on neurological examination, physical examination, and imaging findings [8, 9]. If cervical myelopathy is suspected on physical and neurological examination, cervical spine radiography will be performed to evaluate the presence of ossification of the posterior longitudinal ligament or other degenerative bone changes [10]. The cervical spine consists of seven vertebrae and is referred to as C1–C7 from the skull down; therefore, C1 and C2 refer to the first and second cervical vertebrae from the skull (Fig. 1). A cervical spine radiograph examines the static parameter of the “anteroposterior diameter of the spinal canal” in the lateral view of the cervical spine and the dynamic parameter of the “intervertebral movement of the cervical vertebrae” calculated by comparing the lateral views of cervical anteversion and retroversion [11, 12]. If the anterior–posterior diameter of the spinal canal is narrow and the intervertebral movement is significantly larger than normative values, it is important to understand the cause of spinal cord compression and to decide whether surgical procedure is suitable or not, along with the further evaluation by cervical magnetic resonance imaging (MRI) [13]. However, measuring the dynamic parameter of "intervertebral movement" requires time-consuming methods such as superimposition of X-ray images and calculation by writing many lines [14], which are often not performed in actual clinical practice due to insufficient time.

With recent developments in image analysis technology and machine learning, automatic measurement technology has become increasingly popular [15, 16]. In this study, to reduce the burden on physicians, a system that automatically recognizes the vertebral body parts in lateral images of cervical spine X-ray images and automatically measures intervertebral movements was developed by comparing the cervical anterior and posterior bending images. The accuracy of the system was verified by comparing its results with those of a spine specialist, and its usefulness was examined.

## Materials and methods

### Study participants and input image

Medical records of patients admitted to the Department of Orthopedics and Spine Surgery at Tokyo Medical and Dental University Hospital between May 2013 and September 2015 were retrospectively studied. This study included the records of 484 patients aged between 20 and 100 years. Patients with a history of cervical surgeries or trauma were excluded. A two-way radiograph of the lateral aspect of the cervical spine in full flexion was taken in all patients, and full extension positions were taken by expert radiology technicians; all of which were included in the study.

**Fig. 2:**
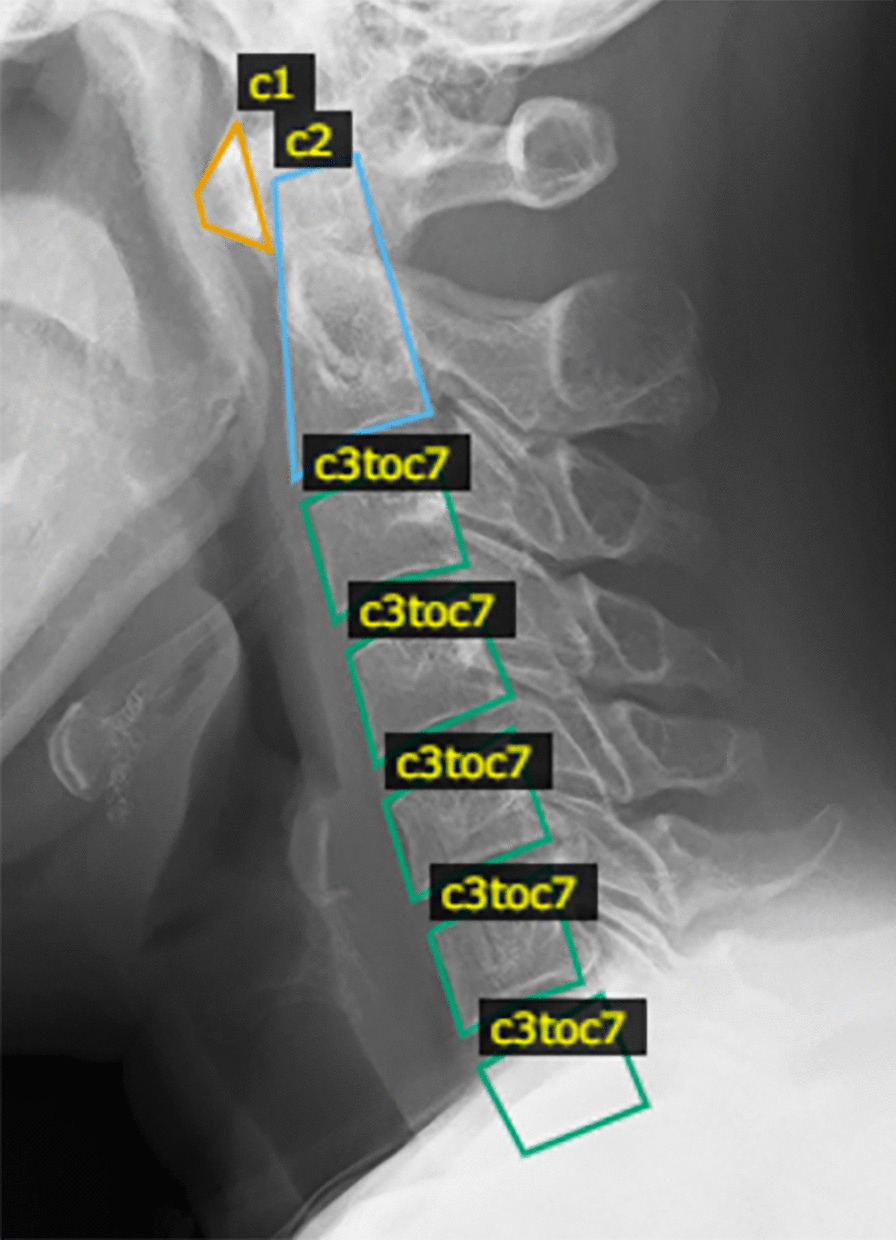
Annotation area for learning model. **c1**, the anterior arch region. **c2**, the region that included the entire vertebrae from the vertebral body to the odontoid process. **c3toc7**, the regions from c3–c7

Digital images of cervical spinal X-ray images were exported in JPEG format. The dataset used in this study consisted of 968 X-ray images of the cervical spine in the flexion and extension positions. Of these 968 images, 922 (461 patients) were used as training data, and the remaining 46 (23 patients) were used as test data. The test data (46 images from 23 patients) were randomly chosen from 484 patients. All X-ray images were labeled according to the guidance of expert spine surgeons (Fig. 2). The cervical spine regions to be masked were different for c3–c7 and for c1 and c2 because of the special shape of the latter. Specifically, the c1 region is the anterior arch of the C1 vertebra, and the c2 region includes the entire vertebrae from the vertebral body to the odontoid process. The regions were labeled “c1”, “c2”, and “c3toc7”.

### System design

#### Overview

**Fig. 3:**
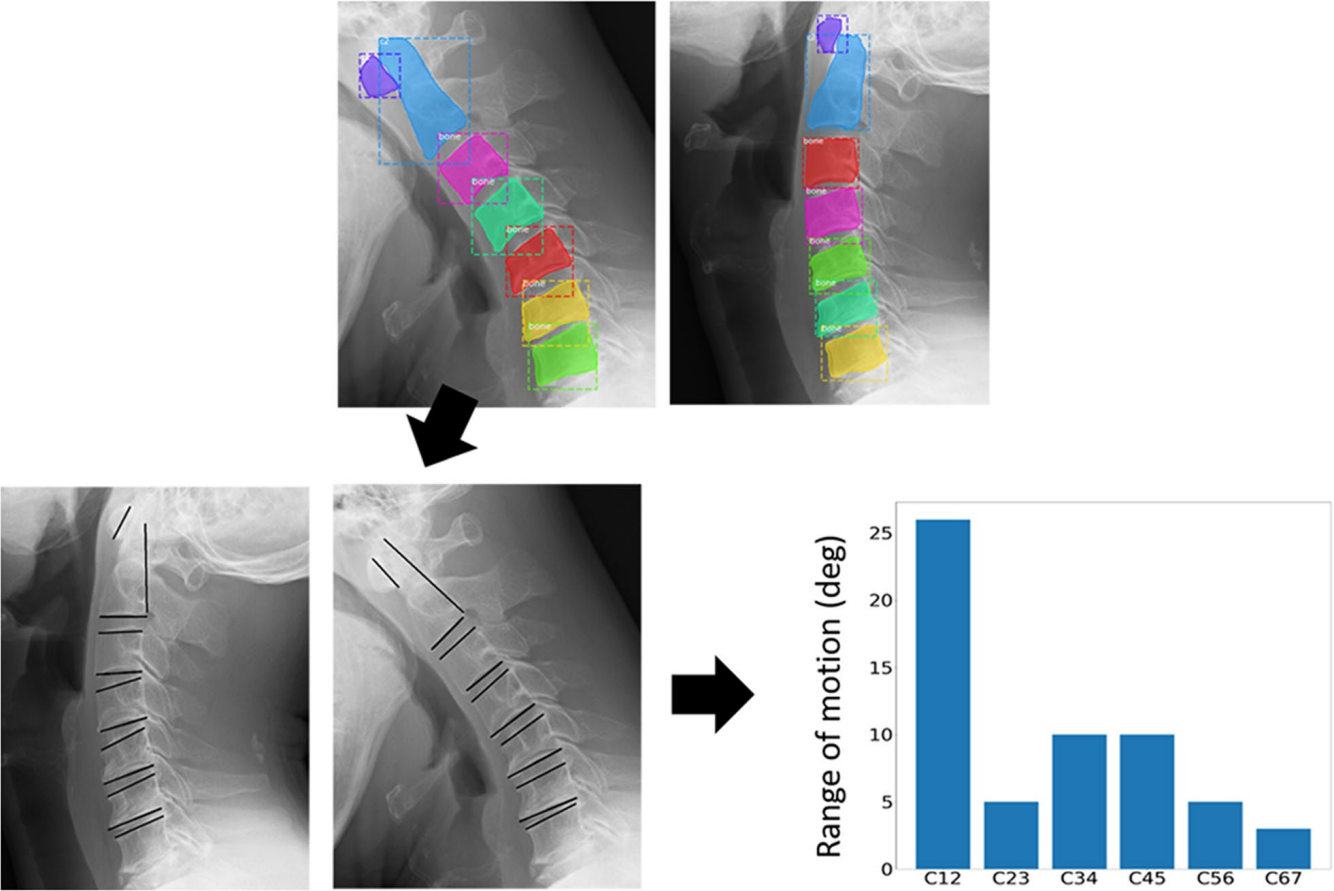
Flow of our proposed system for measurement cervical range of motion (CRoM)

A desktop application for measuring cervical range of motion (CRoM) was developed. Figure 3 illustrates the flow of the proposed system. First, the cervical vertebrae region was estimated from the cervical spine X-ray image using Mask Region-based convolutional neural network (R-CNN) [17]. The estimated region is approximated using a simple polygon with three or four vertices. The edges of the polygons of cervical vertebra n and cervical vertebra n + 1, which are close to the center of each other, are the edges that lie between the cervical vertebrae. The CRoM angle was calculated by measuring the angle between the cervical spine in the flexion and extension images, and then calculating the difference. The displayed angle of the CRoM measurement system was calculated by rounding down to the nearest whole number. The reason is that RoM angles are small and values after the decimal point do not affect diagnosis.

#### Mask R-CNN model construction

Three-class classification and segmentation models were created by using an open-source Mask R-CNN (https://github.com/matterport/Mask_RCNN). The weights learned by Microsoft COCO [18] as the initial weights were used and updated by retraining the entire network using the created training data. The training parameters are listed in Table 1. The training data were divided so that the ratio of training data to validation data was 8:2.

**Table 1:** Hyperparameters in this study

Parameters	
Training data	756 images
Validation data	166 images
Classification	Three-class classification
Number of epochs	100
Image size	512 × 512 pixels
Batch size	1
Learning coefficient	0.001
Optimization method	Stochastic gradient descent

#### Algorithm

**Fig. 4:**
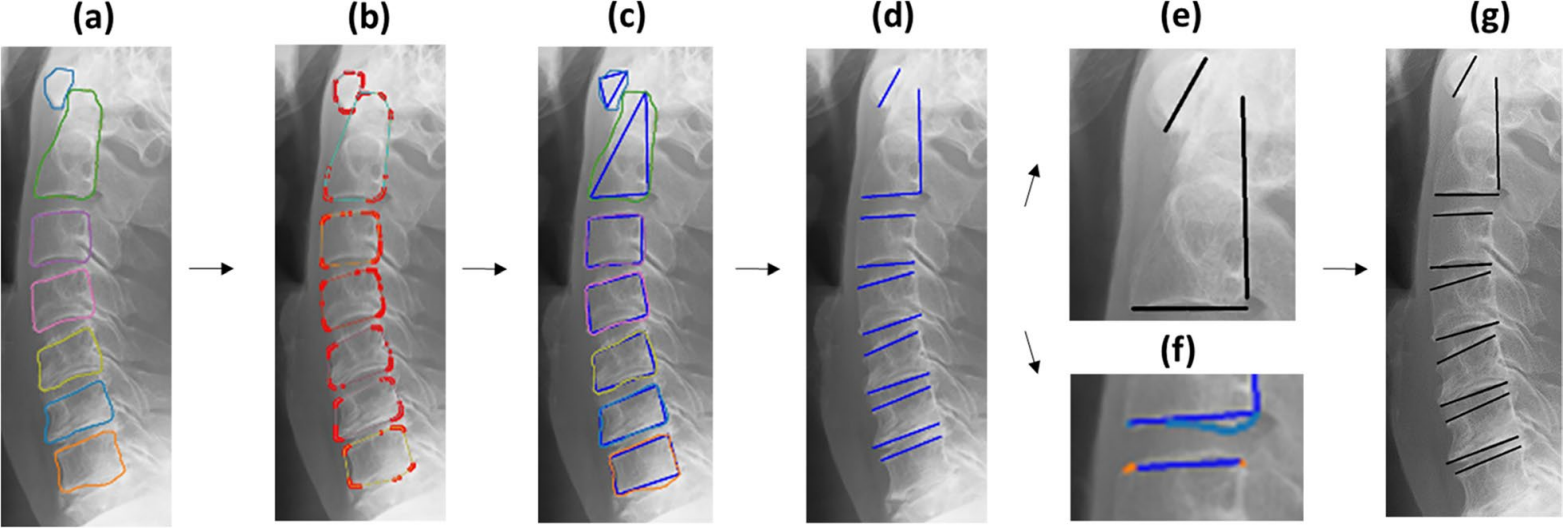
Flow of automatic annotation for detecting the upper and lower edges of the cervical spine. **a** Contour lines of the detecting region. **b** Coordinates of the detecting region. **c** Approximating a convex hull. **d** Selecting the vertebral edge to draw a line. **e** Annotating between C1 and C2. **f** Extraction of the contour coordinates near the selected edge. **g** Annotating between C1/2 and C6/7

**Fig. 5:**
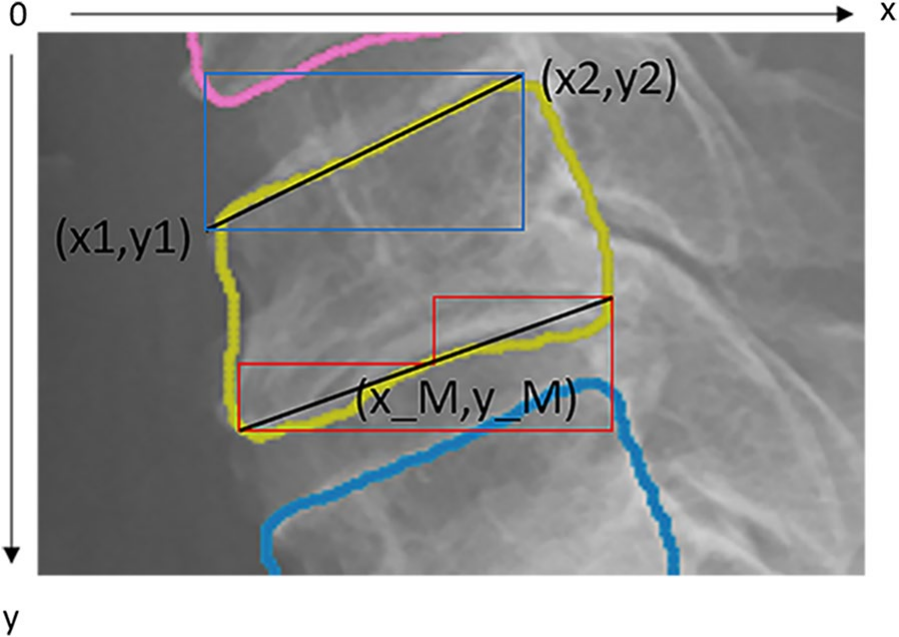
Extraction range of coordinates through which the line passes. × **1**, rightmost coordinate of the edge. **y1**, rightmost coordinate of the edge. × **2**, leftmost coordinate of the edge. **y2**, leftmost coordinate of the edge. **x_M**, midpoint coordinate of the edge. **y_M**, midpoint coordinate of the edge

After the training was completed, a learning model, trained on 378-person images, was saved. At the time of measurement, the Region Proposal Network (RPN) in the learning model saved was used to extract candidate regions of objects in images inputted into the learning model saved, and features were extracted from the boxes of each candidate region using RoIPool [19]. In the training model, class classification and bounding box regression were performed, and object regions were extracted from the input image by performing pixel-by-pixel class classification [17]. The cervical vertebrae are extracted from the image using this principle, and CRoM was measured using the algorithm described below. The bounding box coordinates and area information of the regions estimated by Mask R-CNN are stored in an array but are not arranged in the order of c1–c7. Therefore, it is necessary to shift the cervical vertebrae positions of the estimated regions in both images such that they correspond. To prepare for the measurement, the y-coordinates of the bounding box should be in ascending order, and the estimated regions should be rearranged in the order of c1–c7. However, it is sometimes impossible to estimate some cervical regions in an image. In such a case, the difference between the upper left and lower right y-coordinates of the rectangles of cervical vertebra n and cervical vertebra n + 1 was taken and considered continuous if it is less than half the height of the circumscribed box of cervical vertebra n. If this condition was not met, it was judged that there was a misestimated region or cervical vertebrae region that cannot be estimated. The flow of automatic annotation is shown in Fig. 4. First, the contour coordinates of the region are obtained from the estimated cervical vertebrae region (Fig. 4a). The coordinates of the convex hull are selected from the contour coordinates, and the convex hull region is approximated as a polygon with three or four vertices (Fig. 4b, c). Subsequently, the midpoint of each edge of the approximated polygon is calculated. The edge where the calculated midpoints were close to each other (between cervical vertebra n and cervical vertebra n + 1) was regarded as the edge of the cervical spine used for measurement (Fig. 4d). However, between c1 and c2, the line for measurement is drawn vertically. The algorithm for drawing the line is different from that for below c3. c1 and c2 are approximated as triangles by the algorithm. For this reason, the longest side of the triangle to be approximated is used as the line for measurement for c1, and the line to the right of the lower edge of c2 is used as the line for measurement for c2 (Fig. 4e). Because polygons below c3 are approximated to be inscribed in the convex hull region, their edges may be far from the contour coordinates of the estimated region. In this case, the contour coordinates near the selected edge of the approximate polygon were extracted (Fig. 4f). Then, × 1 and y1 were the coordinates of the left end of the selected edge in the approximate polygon, × 2 and y2 were the right ends, and x_M and y_M were the midpoints (Fig. 5). Let the x-coordinate group of the contour coordinates be verts_x and the y-coordinate group be verts_y. The upper end of the vertebra of interest is the contour coordinate that satisfies the condition of Eq. 1 and is the contour coordinate in the blue box in Fig. 5. The lower edge is the contour coordinate that satisfies the conditions in Eq. 2, which is the contour coordinate in the red box in Fig. 5. A line was drawn on these coordinates using the least-squares method to determine the angle between them (Fig. 4g). The angle between the two lines was calculated from Tangent’s additive theorem using Eq. 3, where a and b are the slopes of the two lines.

1 $$\begin{array}{*{20}c} {\left( {x1 \le verts_{x} \le x2} \right) \cap \left( {y2 \le verts_{y} \le y1} \right)} \\ \end{array}$$

2 $$\begin{array}{*{20}c} {\left\{ {\begin{array}{*{20}l} {\left( {x1 \le verts_{x} \le x_{M} } \right) \cap \left( {y_{M} \le verts_{y} \le y1} \right)} \hfill \\ {\left( {x_{M} \le verts_{x} \le x2} \right) \cap \left( {y2 \le verts_{y} } \right)} \hfill \\ \end{array} } \right.} \\ \end{array}$$

3 $$\begin{array}{*{20}c} {\tan \theta = \frac{a - b}{{1 + ab}}} \\ \end{array}$$

Our system was implemented using Python (version 3.6.12, Python Software Foundation, Delaware, USA), a machine learning library TensorFlow (version 1.13.1, tensorflow.org), a machine learning library Keras (version 2.0.8, keras.io), h5py (version 2.10.0, The HDF Group, Illinois, USA), scikit-image (version 0.17.2, scikit-image.org), scipy (version 1.5.4, scipy.org), matplotlib (version 3.3.3, matplotlib.org), Pillow (version 8.0.1, python-pillow.org), and opencv-contrib-python (version 4.4.0.46, opencv.org).

#### Mask R-CNN model performance evaluation

A total of 756 X-ray images were trained on a Mask R-CNN model for 100 epochs. The test data (46 images from 23 patients) were randomly chosen from 484 patients. The accuracy of the detection in cervical spine regions was evaluated using intersection over union (IoU), which is the similarity between two sets [20]. The IoU value was obtained by dividing the common part of the correct and estimated regions by the union of the two regions, where the maximum value was represented by 1. The higher the value, the higher the accuracy of object detection.

### Manual measurement method

To verify the accuracy of the developed system, manual measurements were performed. On the X-ray images, lines were drawn at the upper and lower edges of each vertebra [12]. The angles between the two lines were measured during flexion and extension. The difference between the two angles was then calculated. Because of the special shapes of C1 and C2, a line was drawn on the posterior margin of the anterior arch for C1 and on the posterior margin of the vertebral body for C2, and the angle between these lines was determined (Fig. 1). These angles were defined as the CRoM.

### Evaluation of accuracy in this system

The accuracy of the automatic measurement method was evaluated by comparing the average error between the true value and the automatic measurement value and between the true value and the resident’s measurement value using the specialist’s measurement as the true value [21]. A specialist in this context refers to a spine surgeon who has specialized in spine surgery for more than 10 years, while a resident refers to a surgeon whose career is less than 3 years. The true values were measured using a homemade system that performed the same angle calculations as the automatic measurement system. Therefore, the angular readings of the true values were made on the same basis. Residents were also asked to take measurements using the same homemade measurement system. The data to be measured were the same 46 images of the test data (23 patients) used to validate the learning model in the cervical region. These images were not included as validation data in Table 1. The true value was measured 69 times by two specialists: 23 persons (test data) × three times (number of measurements). The frequency of measurements was limited to once per day and was not continuous. The true value was the average of three measurements taken by the two specialists. Two residents, who were given guidance on the cervical CRoM angle measurement by a specialist, were asked to measure the test data under the same conditions as that of the specialist.

The error between the true and automatic measurement values was calculated by averaging the difference between the two values for each vertebra as an absolute value. The error between the true value and the value measured by a resident was calculated by averaging the difference between the true value and the resident’s measurement between each cervical spine for each of the three times the resident performed the measurements.

### Statistical analysis

A two-sided t-test at the 5% significance level was performed to determine whether the differences were statistically significant [22]. Differences were considered statistically significant at a P*-*value of ≤ 0.05. Analyses were performed using Microsoft Excel 2016 (Microsoft, Washington, USA). Variable 1 was the error value of the resident, and the number of samples was 798: resident (two persons) × number of measurements (three times) × 133/138 cervical intervals. Variable 2 was the error value of the automatic measurement, and the number of samples was 798: 133/138 cervical spine × the number of measurements of the system (six times).

## Results

### Learning model for the cervical region

Forty-six images of the test data were estimated. Of a total of 322 cervical vertebra in these 46 images, 313 were successfully estimated, meaning that 97% of the total test data could be detected. The IoU values for each cervical spine vertebra are listed in Table 2. The mean IoU value for all the 46-test data was 0.85.

**Table 2:** Intersection over union (IoU) of each cervical spine

Position	IoU
C1	0.74
C2	0.83
C3	0.88
C4	0.88
C5	0.87
C6	0.86
C7	0.86
Average	0.85

The IoU value was used to evaluate the accuracy of detection in cervical spine regions. The maximum value was represented by 1. The higher the value, the higher the accuracy of object detection

*IoU* intersection over union

### Accuracy comparison between the proposed system measurements and the resident’s measurements

**Fig. 6:**
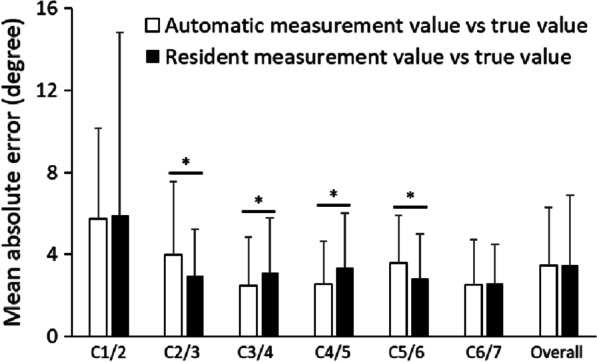
Comparison of the mean absolute error between the resident’s and automatic measurements. There is a significant difference between C2/3 and C5/6, and the resident has a smaller error than that of the automatic measurement. C3/4 and C4/5 also have a significant difference, but the automatic measurement has a smaller error than that of the resident’s measurements. There was no difference in the overall mean error between the automatic and resident’s measurements. *P < 0.05

In the measurement of the CRoM angle in the test data, the number of places where automatic measurement was possible was 133 out of 138 places for 23 people × six (places where the CRoM angle was measured). The remaining five places were not measured because the cervical region could not be segmented. The mean error between the true value of the CRoM angle between each vertebra in the automatic and resident measurements is shown in Fig. 6. The errors between the true value and the automatic measurement were compared with the errors between the true value and the resident's measurement. Figure 6 shows a comparison of the average error between the resident’s and automatic measurements. There was no significant difference in the average error between the resident’s measurements and the automatic measurements. Significant differences in error were examined for C1/2–C6/7. There was a significant difference between C2/3 and C5/6, and the resident’s measurement had a smaller error as compared to that of the automatic measurement. The difference between C3/4 and C4/5 were also significant, but the automatic measurement had a smaller error as compared to that of the resident’s measurement. There was no difference in the overall mean error between the automatic and resident’s measurements. However, the error variance was smaller for the automatic measurement. The standard deviations for each measurement by specialist and residents are shown in Table 3. In the automatic measurement, the standard deviation was zero because the same value was obtained even after three measurements.

**Table 3:** Standard deviation of the specialist’s and resident’s measurements

Vertebra	Specialist value (°)	Resident value (°)
	Standard deviation	Standard deviation
C1/2	4.4	5.2
C2/3	2.4	2.6
C3/4	2.9	2.9
C4/5	2.5	3.0
C5/6	2.7	2.7
C6/7	2.4	2.7
Average	2.9	3.2

### Accuracy comparison between the proposed system measurements and the specialist’s measurements

The mean error between the true value of the CRoM angle between each vertebra in the automatic measurements is shown in Table 4. C3/4, C4/5, and C6/7 had mean errors of less than 3°.

**Table 4:** The mean absolute error and standard deviation of the specialist’s and automatic measurements

Vertebra	Mean absolute error (degree)	Standard deviation (degree)
C1/2	5.7	4.4
C2/3	4.0	3.6
C3/4	2.5	2.4
C4/5	2.6	2.1
C5/6	3.6	2.3
C6/7	2.5	2.2
Average	3.5	2.8

## Discussion

In this study, a system was developed to automatically measure the range of motion of the cervical vertebrae using lateral images of cervical spine X-ray images and verified its accuracy. As a result, it was possible to measure the range of motion with an accuracy equivalent to or better than that of an orthopedic resident. The measurement, which normally takes approximately 3–5 min, can be performed within 10 s using a PC with an Intel Core i7-8565U CPU at 1.80 GHz (Additional file 1: Video), which is expected to greatly contribute to reducing the workload of the physicians. It would be especially meaningful for general physicians with no training in orthopedics to be able to perform screening as accurately as orthopedic surgeons in a short time.

Automatic image analysis technology has been widely studied and applied for the detection of cancer and fractures [23–26]. The image analysis technology and machine learning used in this study are unconventional in terms of the combination of these technologies and have the advantage that the measurement results do not change regardless of how many times the same data are used. It also demonstrated success in extracting important information as an indicator of medical care. The standard deviation of the error of C2/3–C6/7 was not much different from that of the measurements made by the specialists and residents; therefore, it can be used in actual clinical practice and can be applied as an auxiliary system for diagnosis. However, there is a disadvantage in that the measurement accuracy of the movable angle of C1/2 is low, which may need to be solved using another cervical segmentation method.

Automatic image analysis technology in the medical field aims to reduce the workload of physicians and the disadvantages of patients by reducing the number of events missed by physicians, for which the positive rate is relatively low, and is expected to be widely used in the future [27, 28]. However, the direction of this study differs from existing studies in that it aims to reduce the workload of physicians and supplement their lack of experience by aiming to automate the measurement. The increased movement between the cervical vertebrae suggests that the intervertebral disc tissue has begun to degenerate or be damaged, and braking between the vertebrae has begun to fail, which may lead to nerve root or spinal cord compression due to disc herniation [29–31]. MRI is the standard method of evaluating spinal cord compression. A skilled physician can empirically evaluate intervertebral movement without the need for imaging measurements. However, inexperienced or non-specialized physicians need time to measure, given that not much effort is put into this in actual clinical practice. Quantitative evaluation of cervical spine mobility with a system such as this one will assist physicians in saving their time by avoiding manual measurements and help to understand the cause of spine compression.

This study had some limitations. First, the actual degree of spinal cord compression was not analyzed. Because the samples in this study were retrospectively collected and neurological examination and MRI were not performed in all patients who underwent cervical X-ray, the relation among the neurological finding, range of motion and degree of spinal cord compression could not be examined. It is planned to analyze neurological symptoms in patients who have undergone both X-ray and MRI. Second, because this was a single-institution study, selection bias could not be eliminated. In the future, the addition of training data from other hospitals and other cervical segmentation methods will be considered to improve the accuracy of the measurements. Moreover, this system will be incorporated into an image viewer and widely applied to hospitals.

## Conclusions

A system for measuring range of motion of each cervical vertebra on X-ray images was developed and showed accuracy comparable to that of spine surgeons. This system will be effective in reducing the burden on and saving time of orthopedic surgeons by avoiding measuring X-ray image manually.

## Supplementary Information

**Additional file1**. **Video**: Demo of measuring motion at the cervical spine. Automatic measurement of range of motion using cervical spine X-rays within 10 seconds.

## Data Availability

The data that support the findings of this study are available on request from the corresponding author KF. The data are not publicly available due to containing information that could compromise research participant consent.

## Abbreviations

MRI: Magnetic resonance imaging
CRoM: Cervical range of motion
R-CNN: Region-based convolutional neural network
IoU: Intersection over union
